# The role of Notch signalling and its crosstalk with oestrogen receptor signalling in breast cancer

**DOI:** 10.1007/s10555-026-10331-4

**Published:** 2026-03-27

**Authors:** Aygun Azadova, Orkhan Isayev, Antonio Marco, Greg N. Brooke

**Affiliations:** 1 School of Life Sciences, University of Essex, Wivenhoe Park, Colchester, CO4 3SQ UK; 2 Department of Cytology, Embryology, and Histology, Azerbaijan Medical University, Baku, AZ1022 Azerbaijan

**Keywords:** Notch, Oestrogen receptor, Breast cancer, Endocrine resistance, Endocrine therapy, Notch inhibitors, Crosstalk

## Abstract

Breast cancer (BCa) is the most frequently diagnosed malignancy in women worldwide, with approximately 70% of cases driven by oestrogen receptor alpha (ERα). Endocrine therapies aim to suppress ERα signalling activity and form the foundation of current therapeutic strategies. However, a substantial proportion of patients either fail to respond due to intrinsic resistance or acquire resistance over the course of the treatment. This resistance arises through a complex interplay of factors including crosstalk with other signalling pathways such as Notch. Notch signalling, essential for mammary gland development, is aberrantly activated in breast tumours, where it contributes to cancer stem cell maintenance, epithelial-mesenchymal transition, angiogenesis, and metastasis. Notch receptors exert context- and subtype-specific roles: Notch1 and 4 promote tumour aggressiveness, whereas Notch2 often exhibits tumour-suppressive roles. In ERα-positive BCa, ERα and Notch signalling cooperate to drive resistance, whereas in ERα-negative disease, Notch promotes stemness and angiogenesis. While anti-oestrogen therapies effectively inhibit tumour growth, they can paradoxically activate Notch signalling and promote therapeutic resistance. Co-targeting Notch alongside endocrine therapy has been proposed as a strategy to delay the onset of therapeutic resistance. However, clinical development of Notch inhibitors has been limited by toxicity associated with pan-Notch blockade. More selective approaches, such as paralogue-specific antibodies, transcription-complex disruption, rational drug combinations, and advanced delivery platforms, are under active development to overcome these limitations. This review outlines the ERα-Notch crosstalk in BCa and examines current and emerging strategies for targeting Notch to overcome endocrine resistance and improve clinical outcomes.

## Introduction

Breast cancer (BCa) remains the most frequently diagnosed malignancy in women and a leading cause of cancer mortality globally, with 2.3 million new cases in 2022, accounting for 11.6% of all cancers [1]. Most BCa cases are driven by oestrogen receptor alpha (ERα), which promotes tumour growth and survival [2]. Endocrine therapy is given to hormone receptor-positive BCa patients to reduce ERα signalling activity by blocking ERα or by decreasing oestrogen production. Ovarian suppression treatment, ant-ioestrogens (selective oestrogen receptor modulators and selective oestrogen receptor degrader), and aromatase inhibitors are currently administered endocrine therapies in BCa patients with ERα-positive disease [3]. However, clinical benefit is limited by resistance; over 30% of patients exhibit intrinsic non-responsiveness, and many who initially respond relapse with acquired resistance [4]. Resistance arises through diverse mechanisms. These include alterations in ERα itself (*ESR1* mutations, splice variants, or loss of expression), dysregulation of ERα co-regulators, activation of alternative growth factor pathways (HER2, EGFR, IGF-1R) with downstream PI3K/AKT/mTOR and MAPK/ERK cascades, cell-cycle deregulation (Cyclin D1 amplification, p16 loss), and crosstalk with developmental pathways such as Wnt/β-catenin, NF-κB, Hedgehog, and Notch [5].

The Notch signalling pathway plays a crucial role in mammary gland development, regulating stem cell maintenance, lineage specification, and epithelial homeostasis. While Notch is oncogenic in many BCa contexts, its function is context- and isoform-dependent across tissues; when dysregulated in specific oncogenic contexts, Notch contributes to luminal differentiation, epithelial transformation, metastasis, and therapeutic resistance [6, 7]. In ERα-negative tumours, Notch receptor signalling is often upregulated and strongly linked to tumour aggressiveness [8, 9]. In ERα-positive disease, inhibition of ERα or oestrogen deprivation has been shown to paradoxically increase Notch activity, implicating the pathway in endocrine resistance [10]. Notch also interacts with major oncogenic cascades, including Wnt, Hippo, PI3K/AKT, Hedgehog, MAPK/ERK, and others, thereby amplifying proliferative and survival signals [11]. Given its central role at the intersection of developmental processes and therapeutic resistance, Notch represents a promising therapeutic target. Therapeutically, multiple strategies to inhibit Notch signalling have been investigated, such as γ-secretase inhibitors that block Notch activation, receptor- or ligand-specific antibodies to block signal initiation, small molecules like CB-103 that disrupt the Notch transcription complex, and natural compounds [12].

This review aims to provide a comprehensive understanding of ERα–Notch crosstalk in BCa and to highlight current strategies for targeting the Notch pathway. We first provide an overview of the Notch and ERα signalling pathways and then examine the context-dependent roles of Notch receptors across different BCa subtypes, with particular emphasis on their interactions with ERα signalling in hormone receptor-positive disease. Finally, we explore current and emerging therapeutic strategies targeting the Notch pathway.

## Oestrogen receptor signalling

**Fig. 1:**
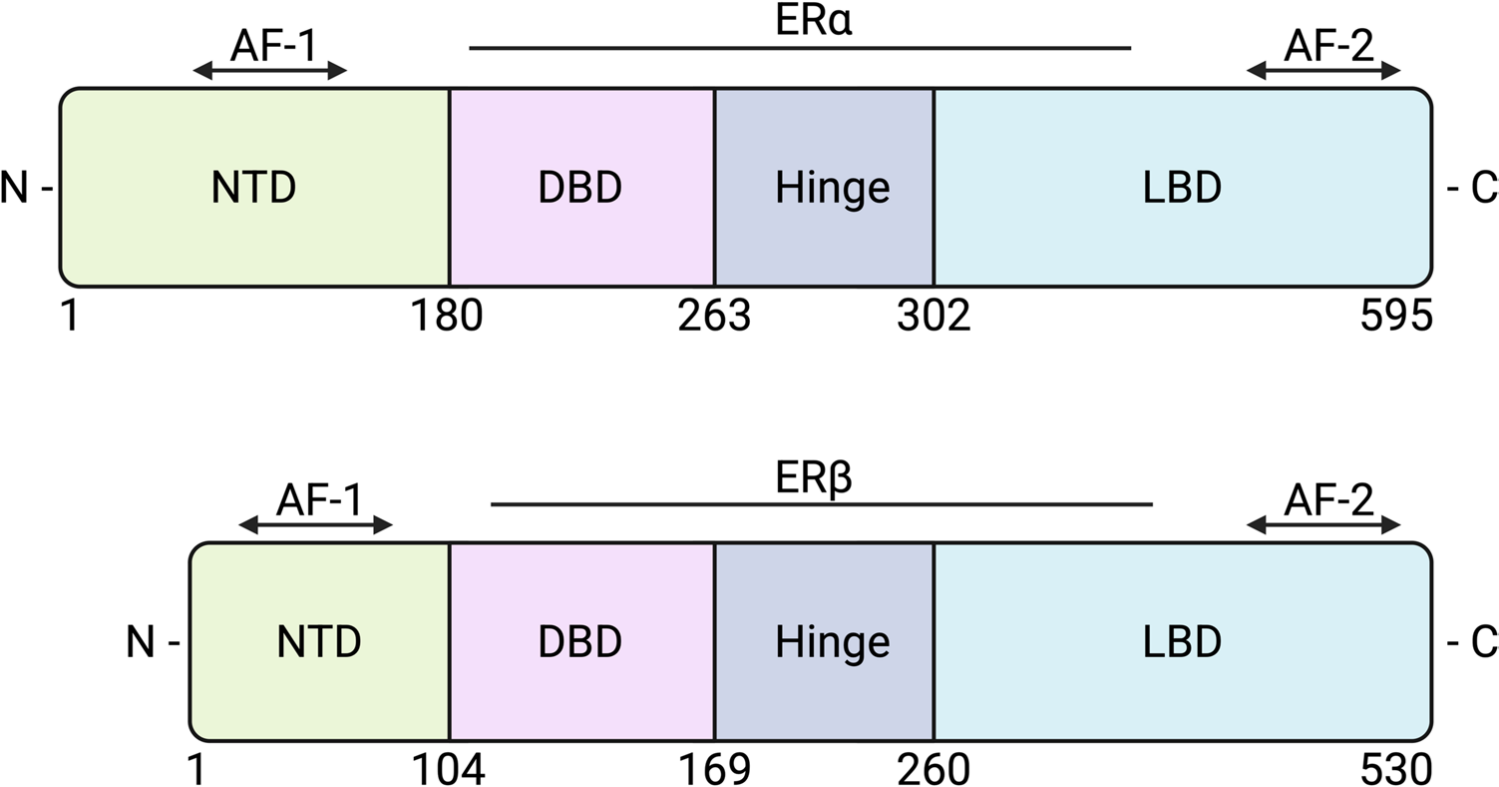
Domain architecture of oestrogen receptor α and β. The schematic illustrates the structural organisation of human ERα and ERβ proteins. Both receptors share a modular structure consisting of the N-terminal domain (NTD), DNA-binding domain (DBD), hinge region, and ligand-binding domain (LBD). The activation function-1 (AF-1) is located within the NTD and is constitutively active, while activation function-2 (AF-2) is located within the LBD and is ligand-dependent. ERα (595 amino acids) and ERβ (530 amino acids) differ in the length and sequence of their domains, which influence their transcriptional activity and response to oestrogens and selective modulators. The figure was created using biorender.com

Oestrogens are the primary female sex hormones and are endogenously synthesised in the form of oestrone, 17β-oestradiol, oestriol, oestetrol, and 27-hydroxycholesterol and act through the oestrogen receptors, ERα and ERβ and a membrane G protein-coupled oestrogen receptor (GPER1/GPR30) [13]. They play an essential role in regulating reproductive function, menstrual cyclicity, fertility, skeletal integrity, glucose metabolism, immune function, and the development of secondary sexual characteristics [14]. ERα and ERβ have a modular structure that consists of common domains: N terminus, DNA-binding domain, hinge region, ligand-binding domain and C terminus. The large amino-terminal domain contains a hormone-independent transcriptional activation function 1 (AF1), where coactivator proteins bind. The ligand-binding domain also contains another activation function surface (AF2) that facilitates ligand-dependent transcription by binding to coregulatory factors. The ER receptors are encoded by distinct genes: *ESR1* (ERα) on chromosome 6q25.1 and *ESR2* (ERβ) on 14q23.2. *ESR2* encodes a 530-amino acid protein (~ 59 kDa), whereas ERα encodes a slightly larger 595-amino acid protein (~ 67 kDa), with both receptors organised into eight exons (Fig. 1) [15]. GPER1 is structurally unrelated, consisting of seven transmembrane helices with four extracellular and four intracellular domains [16].

In the normal mammary gland, ERα is mainly confined to luminal epithelial cells, while ERβ is distributed across luminal, myoepithelial, and stromal cells, making ERβ expression more widespread [17]. Nevertheless, knockout mouse models show that mammary gland development is critically dependent on ERα but not ERβ [16]. Both receptors exist as multiple isoforms due to alternative splicing and the use of different translation start sites. ERα variants include ERα66, ERα46, ERα36, ERαΔ3, ERαΔ5, and ERαΔ7, while ERβ isoforms comprise ERβ1–5 and ERβΔ3 [18]. Notably, ERβ2–5 have limited or no capacity to bind oestrogen. Sequence analysis reveals ~ 97% amino acid identity in the DNA-binding domain of ERα and ERβ and moderate similarity (~ 59%) in the ligand-binding domain and AF2, but considerable divergence in AF1 [19].

**Fig. 2:**
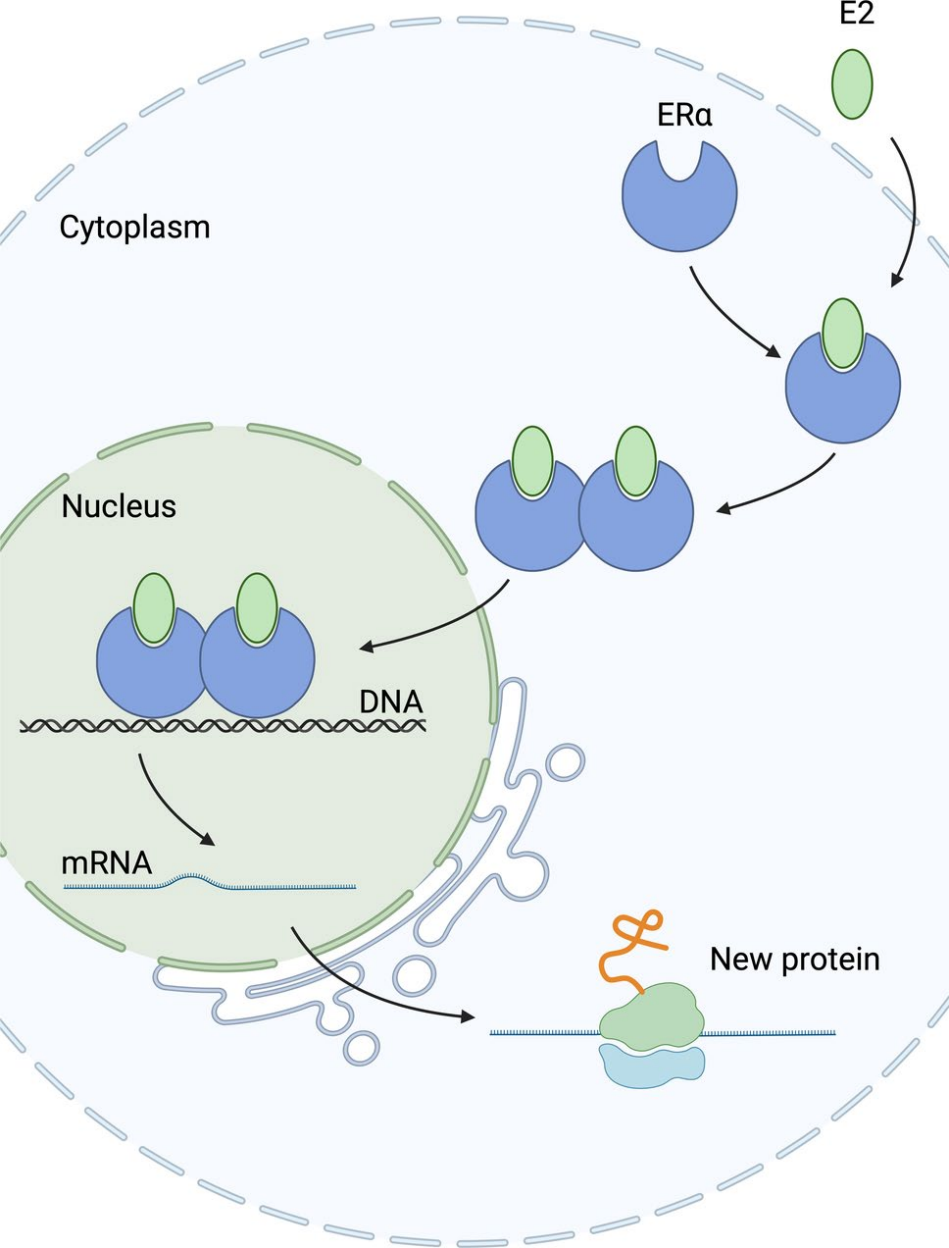
Canonical oestrogen receptor α signalling pathway. Schematic illustrates the classical genomic signalling mechanism of oestrogen receptor alpha (ERα) in response to 17β-oestradiol (E2). Upon E2 binding in the cytoplasm, ERα undergoes a conformational change, dimerises, and translocates to the nucleus. The ERα-E2 complex binds to specific oestrogen response elements (EREs) in the DNA, initiating transcription of target genes. The figure was created using biorender.com

Oestrogen receptors signal through both genomic and non-genomic pathways. In the canonical genomic pathway, ligand binding to ERα or ERβ induces conformational change, receptor dimerisation, and nuclear translocation, where the complex recruits coactivators such as the SRC/p160 family and CBP/p300 to bind oestrogen response elements (EREs) in DNA (Fig. 2). Over 70,000 EREs have been identified within the human genome, often near promoters or enhancers, thereby regulating transcription. Importantly, chromatin immunoprecipitation sequencing has shown that ERα also occupies genomic regions lacking classical EREs [14]. Non-classical genomic signalling occurs via protein–protein interactions with transcription factors, including AP-1, NF-κB, SP-1, and NF-Y, bypassing direct DNA binding [20]. In contrast, rapid non-genomic signalling is triggered within seconds to minutes when oestrogen interacts with membrane-bound ERs, such as GPER1 and certain ERα/ERβ variants. This leads to activation of multiple intracellular cascades, including the Ras-Raf-MEK-MAPK cascade and the PI3K/AKT signalling axis, acting through the PI3K-AKT-mTOR pathway [21]. Finally, ligand-independent receptor activation can occur when phosphorylation of ER by protein kinases, post-translational modifications, or mutations in the ligand binding domain induce structural changes that activate ER without oestrogen [22].

## Notch signalling

The Notch signalling pathway is an evolutionarily conserved cell–cell communication mechanism that plays a vital role in regulating cell fate determination, lineage specification, differentiation, and the maintenance of tissue homeostasis [23]. The pathway was first described in the early twentieth century, when notched wing phenotypes in *Drosophila melanogaster* were observed to be caused by genetic mutations and later characterised as an allele responsible for that phenotype. The *Notch* gene was subsequently cloned and sequenced in 1980 [24]. Since then, Notch signalling has been recognised as a critical regulator of numerous developmental and physiological processes, and its dysregulation has been implicated in a wide range of pathologies, including congenital disorders, inflammatory diseases, and cancer [11].

**Fig. 3:**
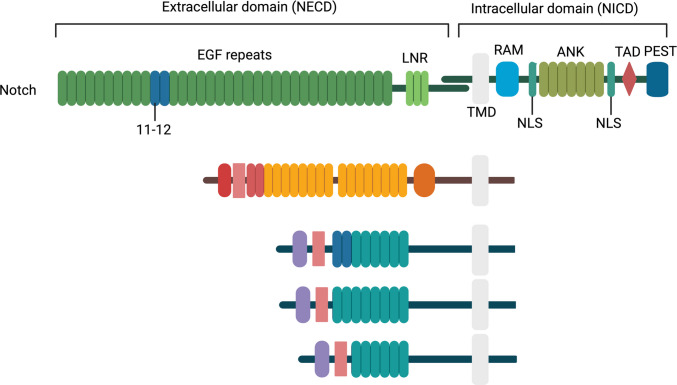
Domain architecture of mammalian Notch ligands and receptors. The schematic illustrates key extracellular domains of human Notch ligands—Jag1/2 and Dll1/3/4—as well as a representative Notch receptor (e.g. Notch1). All ligands share a conserved DSL (Delta/Serrate/Lag-2) domain necessary for receptor interaction. Jag1 and Jag2 uniquely contain a DOS (Delta and OSM-11-like) domain and a cysteine-rich region, which contribute to ligand-specific functions. MNNL domains are present in all except Dll3. The number of EGF-like repeats varies by ligand: 16 in Jag1/2, 8 in Dll1/4, and 6 in Dll3. The Notch receptor extracellular domain is composed of 36 EGF-like repeats, with EGF repeats 11–12 being critical for ligand binding. Additionally, the receptor contains three LIN12/Notch repeats (LNR) and a heterodimerisation domain, which protect the receptor from premature activation. The figure was created using biorender.com

**Fig. 4:**
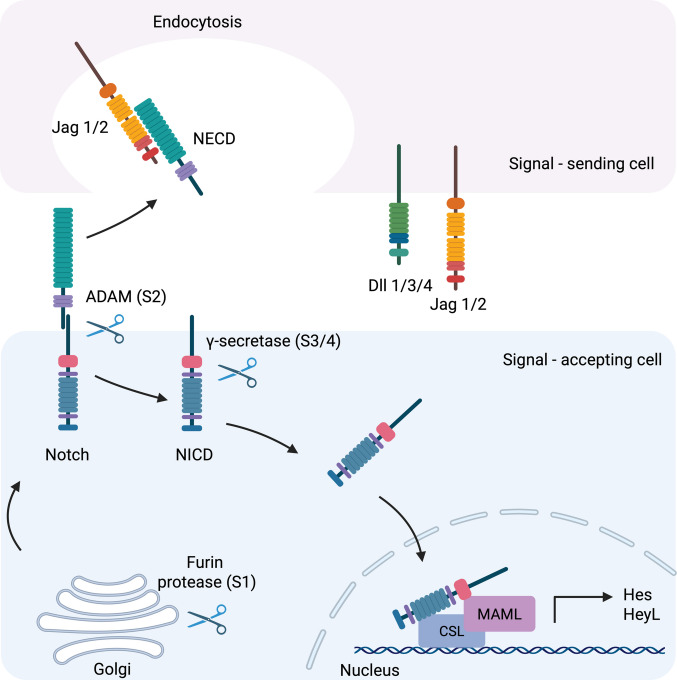
Schematic representation of canonical Notch signalling. Notch signalling is initiated through direct cell–cell interaction between a signal-sending cell expressing a Notch ligand (Jag1/2 or Dll1/3/4) and a signal-accepting cell expressing a Notch receptor. Ligand binding triggers mechanical pulling that exposes the S2 cleavage site in the receptor, allowing cleavage by an ADAM metalloprotease. This is followed by intramembrane cleavage at the S3 site by the γ-secretase complex, releasing the Notch intracellular domain (NICD). Prior to membrane localisation, the receptor undergoes S1 cleavage by furin protease in the Golgi. Once released, NICD translocates to the nucleus, where it associates with the DNA-binding protein CSL (CBF1/Suppressor of Hairless/LAG-1) and coactivator MAML, initiating transcription of Notch target genes such as HES and HEYL. The figure was created using biorender.com

In mammals, the Notch pathway comprises four single-pass transmembrane receptors (Notch1–4) and five transmembrane ligands, including three Delta-like (Dll1/3/4) and two Jagged (Jag1/2) factors which are homologues of the *Drosophila* Serrate proteins (Fig. 3) [7]. The Notch receptors are synthesised as precursor proteins that undergo essential post-translational modifications in the endoplasmic reticulum such as O-fucosylation, O-glucosylation, and O-GlcNAcylation [25]. The modified precursor proteins are then transported to the Golgi apparatus, where they are cleaved by furin protease at the S1 site to yield a heterodimeric complex that includes an extracellular domain (NECD), a transmembrane region, and an intracellular domain (NICD) (Fig. 4). These two domains remain associated as a heterodimer and are transported to the cell surface as a functional receptor [12].

The NECD contains 29–36 EGF-like repeat domains that are responsible for ligand binding, as well as a negative regulatory region (NRR) comprising three Lin12-Notch repeat (LNR) domains rich in cysteine residues, which prevent ligand-independent activation (Fig. 3). The transmembrane region contributes to heterodimer stability through its short extracellular component and conserved cysteine residues that are stabilised by Ca^2+^ ions [26]. The NICD contains RBP-Jk-associated molecule (RAM) domain together with two nuclear localisation sequences (NLS), seven ankyrin repeats (ANK), a transactivation domain (TAD), and a PEST sequence enriched in proline, glutamic acid, serine and threonine residues. The RAM domain facilitates interaction with DNA-binding proteins such as C promoter-binding factor-1 (CBF1), also known as Suppressor of Hairless or Lag-1 (CSL), while the ANK repeat domain mediates the recruitment of the transcriptional coactivator Mastermind-like (MAML) proteins. The PEST domain regulates protein stability and determines the half-life of the Notch intracellular domain (Fig. 3) [11].

Notch ligands contain a Module at the N terminus of Notch Ligands (MNNL) domain followed by a conserved Delta/Serrate/LAG-2 (DSL) domain for receptor binding (interacting with EGF repeat 11,12 in the Notch receptor), and 7–16 EGF repeats (first two EGF repeats in Jag1/2 and Dll1 are called the DOS (Delta/OSM-11 motif)). The Jagged ligands also include unique cysteine-rich and von Willebrand factor C domains, which modulate ligand dimerisation and receptor specificity (Fig. 3) [26].

Activation of the Notch signalling pathway is usually initiated through direct cell–cell contact, whereby a membrane-bound ligand on a signal-sending cell engages a Notch receptor on an adjacent signal-receiving cell. This ligand and receptor interaction triggers a cascade of events that lead to transcriptional regulation of target genes within the nucleus of the receiving cell, a process referred to as trans-activation. In contrast, when Notch ligands and receptors are expressed on the same cell and interact locally, the phenomenon is known as cis-inhibition, which typically serves to modulate or suppress pathway activation [27].

In canonical Notch signalling, upon ligand binding, the Notch receptor undergoes a conformational change in its extracellular domain, which facilitates receptor endocytosis by the ligand-presenting cell. This mechanical pulling force exposes a cleavage site within the NRR of the Notch receptor, allowing an extracellular matrix metalloprotease, typically ADAM10/17, to cleave at the S2 cleavage region, releasing the NECD. This is followed by a second, intramembrane cleavage catalysed by the γ-secretase complex at two distinct sites (S3/4) within the transmembrane domain [28].

Due to its nuclear localisation sites, the NICD subsequently migrates to the nucleus. In the absence of the NICD, the DNA-binding transcription factor CSL (C promoter binding factor-1 (CBF1), Suppressor of hairless, Lag-1) acts as a transcriptional repressor. It does so by recruiting a range of corepressor complexes, including the silencing mediator of retinoid and thyroid hormone receptors, nuclear receptor corepressor, histone deacetylases, and CBF1-interacting corepressors. These corepressor complexes collectively maintain target genes in a silenced state under basal conditions [29].

Upon activation of the pathway and entry of NICD into the nucleus, NICD binds directly to CSL, displacing the repressor complexes and converting CSL from a transcriptional repressor into a transcriptional activator. The CSL is composed of three domains, N-terminal domain (NTD), β-trefoil domain (BTD), and the C-terminal domain (CTD). The CSL-NTD and CSL-CTD regions recognise and bind preferentially to a consensus DNA sequence defined by (C/T)GTGGGAA [30]. This NICD-CSL interaction facilitates the recruitment of MAML1-3 and the formation of this ternary complex (MAML-NICD-CSL) enables the recruitment of additional transcriptional coactivators, including RBPJκ and the p300 histone acetyltransferase complex, resulting in transcriptional activation (Fig. 4) [11].

The Notch signalling pathway also activates gene expression through non-canonical mechanisms that do not involve receptor cleavage by γ-secretase, nuclear translocation of the NICD, or interaction with CSL. One prominent non-canonical route involves the E3 ubiquitin ligase Deltex, which facilitates endocytosis of the Notch receptor in the absence of ligand binding. In this context, Notch can signal from intracellular compartments such as endosomes, bypassing the requirement for surface-level ligand-receptor interactions [28]. The other activation route can be via EGF repeat containing proteins (nnP-1 and MAGP-2, Delta/Notch-like EGF-related receptor and Delta-like 1 homolog) that can bind to and activate Notch receptors [31]. Although the molecular mechanisms underlying non-canonical signalling are less well characterised than those of the canonical pathway, growing evidence suggests that non-canonical Notch activity may crosstalk with other signalling networks, including Wnt, Hippo, PI3K/AKT, TGF-β, Hedgehog, NF-κB, PTEN, HIF1α, IL-6/STAT, IKK, ATM, RAC1, and mTOR, thereby influencing a wide range of cellular outcomes such as stemness, immune regulation, and tumour progression [11].

Among the most well-characterised downstream targets of Notch signalling are members of the Hairy and Enhancer of Split (*HES1-7*) family, the Hairy/Enhancer-of-Split-related with YRPW motif (*HEY1/2*, *HEYL*) family and Notch-regulated ankyrin repeat protein (*NRARP*). These genes encode basic helix-loop-helix (bHLH) transcriptional repressors that play critical roles in regulating cell fate determination, proliferation, and differentiation across various tissues. In addition to these gene families, Notch signalling influences a broad network of transcriptional programmes, including genes regulating angiogenesis (*VEGF*, *PDGFRβ*), cell cycle progression (*CCND1*,* CCND3*,* E2F1*,* CDKN1A*,* CDKN1B*), apoptosis and survival (*BIRC5*,* AKT*,* TP53*,* BCL2*), stemness and pluripotency (*MYC*,* GATA2*,* GATA3*,* BMI1*), epithelial-mesenchymal transition (*SNAI1*,* SNAI2*,* MMP9*,* PTGS2*), immune signalling and inflammation (*IL2R*, *NF-κB*, *MTOR*,* OLFM4*), oxidative stress (*NFE2L2*), cell adhesion (*CD44*), neurogenesis (*REST*), and T-cell development (*PTCRA*,* TCF7*) [32].

## Notch signalling in breast development and breast cancer

The Notch signalling pathway is a fundamental regulator of mammary gland development and homeostasis, playing a critical role in cell fate determination, particularly in epithelial lineage specification [33]. During perinatal development, mammary morphogenesis is driven by bipotent mammary stem cells (MaSC) capable of giving rise to basal and luminal lineages. However, postnatal development is sustained largely by lineage-restricted unipotent progenitors of the luminal and myoepithelial lineages. Active Notch signalling directs progenitor commitment toward the luminal epithelial lineage, with Notch1/2/3 expressed at high levels during luminal differentiation. Knockdown of Notch receptors in MaSC favoured differentiation toward the myoepithelial lineage [7]. Expression profiling revealed that Notch ligands are differentially distributed across mammary epithelial subpopulations: Dll1 is enriched within the MaSC-enriched basal compartment, whereas Jag1 is largely confined to the luminal epithelial lineage [33]. Considering Notch signalling is essential for mammary development, it is unsurprising that aberrant activation of the Notch signalling pathway drives key oncogenic processes, including epithelial-mesenchymal transition (EMT), therapeutic resistance, angiogenesis, uncontrolled proliferation, apoptosis evasion, and metastasis across various BCa subtypes, including hormone receptor-positive disease [34, 35].

An oncogenic effect of Notch was first demonstrated in T-cell acute lymphoblastic leukaemia with the identification of a chromosomal translocation that resulted in the expression of a truncated, constitutively active form of the Notch1 protein [24]. The significance of Notch signalling in BCa was first highlighted by the discovery that the *Notch4* gene is a common site of proviral integration by the mouse mammary tumour virus (MMTV), which leads to the development of mammary adenocarcinomas. Subsequent studies also identified the *Notch1* gene as a potential integration site for MMTV [36]. Notch expression also varies across BCa subtypes; Notch3 is overexpressed in luminal A, while Notch4 is elevated in luminal A and B subtypes. In basal-like BCa, high expression of Notch1/3/4 and Jag1 correlates with increased relapse risk and poor survival [37].

### Notch1

Notch1 is the most extensively studied Notch receptor in BCa with diverse oncogenic roles spanning proliferation, survival, angiogenesis, EMT, therapeutic resistance, and stemness regulation. Hyperactivation of Notch1, marked by NICD accumulation and RBP-Jκ activity, was sufficient to transform normal mammary epithelial cells [6]. In HER2-positive models, Notch1 activation contributes to trastuzumab resistance by suppressing PTEN and hyperactivating ERK1/2, sustaining BCSC survival [9]. Notch1 has also been shown to enhance HER2 expression, contributing to recurrence of HER2/neu-driven tumours, and its inhibition reduces the self-renewal capacity of HER2-positive BCSCs [29]. JNK/c-Jun signalling drives Notch1 activation, which is necessary for the maintenance of the stem cell phenotype in TNBC. In xenograft mouse models and TNBC, knocking down of JNK reduces cell proliferation, ALDH1⁺ and CD44⁺/CD24⁻ BCSC subpopulations, and mammosphere formation, indicating diminished stem-cell-like features [7]. Endothelial Jag1 activates Notch1 in BCSCs, upregulating *ZEB1* and *VEGFA* in a feedback loop that enhances angiogenesis, tumour initiation, and aggressiveness [10]. Notch1 also promotes angiogenesis via Dll4-VEGF-FOXC2 signalling [35].

Notch1 also drives EMT via Jag1-mediated Slug induction and E-cadherin repression, enhancing invasiveness and nodal metastasis [10]. Chen et al. showed that cancer-associated fibroblasts secrete high levels of the extracellular matrix protein, MFAP5, which, when applied to MCF-7 BCa cells, enhances migration, invasion, and EMT through activation of Notch1 signalling and upregulation of Slug [38]. In contrast to studies describing Notch1 as pro-invasive, Zhang et al. found that its activation suppressed BCa collective migration, tumour growth, and metastasis [39].

Clinical studies consistently highlight the prognostic significance of Notch1 in BCa. It is markedly overexpressed in invasive ductal carcinoma than non-invasive tumours [40]. Engel et al. found that in early BCa, Notch1 is present in ~30% of tumours, where high expression associates with hormone receptor negativity, HER2 positivity, shorter recurrence-free interval, and reduced chemotherapy benefit [41]. Analysis of 7,000 BCa transcriptomes, xenograft TNBC models, and immune checkpoint blockade-treated cohorts revealed that combined Notch1/4 overexpression predicts increased recurrence risk in ERα-negative BCa [42]. In a cohort of 115 primary BCa samples, Notch1 positivity was recorded in ~89% of tumours that correlated with higher TNM stage, metastasis, TNBC subtype, and BCSC marker ALDH1 [36].

### Notch2

In contrast to the predominantly oncogenic functions of Notch1, Notch2 has been shown to act mainly as a tumour-suppressive receptor in BCa. Elevated Notch2 expression has been associated with improved disease-free survival, suggesting an antagonistic interaction between Notch1 and Notch2 [43]. In the MDA-MB-231, constitutive activation of Notch2 induced apoptosis and suppressed xenograft growth [36]. Capulli et al. found that Notch2-high BCa cells preferentially enter dormancy within the bone microenvironment, adopting a quiescent, stem-like state associated with better survival outcomes [44]. Notch2 knockdown in TNBC xenografts accelerated tumour growth up to sevenfold, accompanied by enhanced angiogenesis, elevated cytokines, and compensatory Notch1 activation [45]. Clinically, high Notch1 expression correlates with poor differentiation and prognosis, while elevated Notch2 is associated with well-differentiated tumours and favourable survival outcomes [10]. Although most evidence suggests that Notch2 acts as a tumour-suppressive receptor in BCa, a few studies have reported contexts in which it displays oncogenic activity. Notch1/2 overexpression or mutation can drive oncogenesis in TNBC through HES1/HEY1-mediated recruitment of EZH2/PRC2, resulting in PTEN silencing and poor survival [8].

### Notch3

The role of Notch3 in BCa is context-dependent, displaying both oncogenic and tumour-suppressive functions. Oncogenic activity has been reported in HER2-negative tumours, where Notch3 correlates with Dll4 expression and advanced stage and high grade [46]. Constitutive activation of Notch3 promotes proliferation, survival, and EMT, with studies in basal BCas showing ligand-independent activity. Notch3 inhibition reduces growth, whereas agonist stimulation enhances the transformed phenotype and activates oncogenic transcriptional programmes such as *MYC* and *ID4* [24]. In TNBC, Jag1/Notch3 signalling drives angiogenesis and progression, with high expression associated with poor survival [47]. Elevated Notch3 also showed stem-like features (CD44⁺/CD24⁻, ALDH1 activity) and promoted stemness and metastasis in ERα-positive and TNBC models, and its inhibition suppresses self-renewal, invasion, and lung metastasis formation, while partly restoring luminal/ER expression [33]. Mansour et al. reported that BCSC-like populations exhibit significantly elevated PD-L1 expression up to threefold compared to more differentiated cancer cells and demonstrate that this upregulation is mediated via a Notch3/mTOR signalling pathway that contributes to immune evasion in BCa by enhancing PD-L1 on stem-like tumour cells [48].

Emerging evidence also supports a tumour-suppressive function. Notch3-deficient mice show increased tumour initiation, while overexpression suppresses proliferation via HEYL-mediated repression of the main regulator of cell cycle, MYBL2 [49]. Notch3 activation suppresses BCa cell proliferation by inducing G_0_/G_1_ arrest through the Cdh1–Skp2–p27 axis. In MDA-MB-231 cells, overexpression of the Notch3 intracellular domain reduced proliferation and colony formation [10]. Zhang et al. demonstrated that in MCF-7 and T47D, Notch3 knockdown reduced PTEN levels and enhanced proliferation and migration. Contrarywise, overexpression of the Notch3 intracellular domain in MDA-MB-231 increased PTEN, reduced AKT–mTOR pathway activation, and lowered Cyclin D1, resulting in decreased proliferation, invasion, and tumourigenesis [50]. Notch3 acts as a tumour suppressor in BCa by transcriptionally upregulating GSK3b and PTEN, inducing STAT5A, and suppressing ZEB1 (while boosting E-cadherin and reducing vimentin), thereby inhibiting EMT and metastasis, correlating with ERα positivity and improved recurrence-free survival [51, 52].

### Notch4

Notch4 is highly expressed and activated in TNBC, where it functions as an effective marker of mesenchymal-like BCSCs. Compared with other stem-cell markers such as CD24⁻/CD44⁺ or ALDH⁺, Notch4⁺ cells are more invasive and more tumorigenic. Activated Notch4 transcriptionally upregulates Slug to drive EMT and GAS1 to enforce quiescence and cell cycle arrest [53]. Clinically, Notch4 expression is most frequent in TNBC (55.6%) and HER2-positive (45.8%) tumours, but less common in luminal cancers (25.5%). High expression is associated with hormone-receptor negativity, larger tumour size, lymph node involvement, advanced stage, and poorer 5-year overall survival in luminal subtypes [54]. According to Boustan et al., Notch4, nicastrin, and HES1 expressions are significantly upregulated in tamoxifen-resistant ERα-positive BCa patients compared with non-resistant patients. Elevated expression is also associated with worse clinical features such as higher N stage, extracapsular nodal extension, perineural invasion, and nipple involvement [55].

### Dll1/3/4

Sales-Dias and colleagues investigated the oncogenic role of the Notch ligand Dll1 across distinct BCa subtypes. Dll1 depletion in MCF-7 cells markedly reduced proliferation, clonogenic potential, and migration, while inducing G_1_ arrest and apoptosis. In BT-474, Dll1 suppression mainly impaired colony formation, whereas in MDA-MB-231, it reduced migration and invasion without substantially affecting proliferation [56]. Kumar et al. identified a population of Dll1⁺ quiescent tumour-initiating cells within luminal-like BCas that drive tumour growth, metastasis, and chemoresistance. Transcriptomic and chromatin profiling revealed that Dll1⁺ cells are enriched for gene expression signatures of NF-κB signalling, hypoxia response, stem cell-like behaviour, metastatic pathways, and quiescence [57]. Importantly, Dll1 is significantly overexpressed in ERα-positive luminal BCa, where its high expression predicts poor prognosis; this is not observed with other Notch ligands [58].

Other Delta ligands are also clinically relevant. Yuan et al. demonstrated that Dll3 is significantly overexpressed in invasive BCa tissues compared with normal breast tissue. Importantly, Dll3 overexpression correlated with altered immune infiltration, including increased regulatory T cells and expression of immune checkpoint markers such as PD-1 and CTLA-4 [59]. Finally, Dll4 is upregulated in docetaxel-resistant MCF-7 cells compared with controls. Dll4 knockdown sensitised resistant cells to docetaxel, significantly reducing survival and increasing apoptosis [60].

### Jag1/2

Jag1 is a key oncogenic ligand in BCa, with high expression in aggressive subtypes; expression analyses show Jag1 is consistently higher in TNBC and basal-like tumours than in luminal cancers, supporting its role in poor prognosis [61]. Jag1 was found to be significantly overexpressed in BCa tissues compared with adjacent non-tumour tissues, high expression correlating with lymph node involvement, distant metastasis, advanced TNM stage, poor overall survival, and increased recurrence [62]. Jag1 induces EMT by upregulating Slug, repressing E-cadherin, and increasing vimentin expression. The knockdown of Slug reversed these effects [60]. Sun et al. showed that Notch1 and Jag1 proteins are highly expressed in invasive ductal carcinoma and ductal carcinoma *in situ* compared with atypical and usual ductal hyperplasia, with expression strongly associated with lymph node metastasis, advanced TNM stage, and higher pathological grade [63]. Similar to Jag1, Jag2 has been correlated with aggressive BCa phenotypes. In TNBC, it maintains cancer stemness and mediates paclitaxel resistance [64].

### Numb

Numb, a key negative regulator of Notch signalling, is frequently downregulated in BCa, with reduced expression observed in up to 50% of cases. Sabbioni et al. identified that the CRL7-FBXW8 ubiquitin ligase complex targets Numb for proteasomal degradation in BCa. Silencing CRL7-FBXW8 components restores Numb levels, suppresses malignant traits in BCa cell lines, and reduces tumour growth in patient-derived xenografts [65, 66].

## ER-Notch signalling crosstalk

**Fig. 5:**
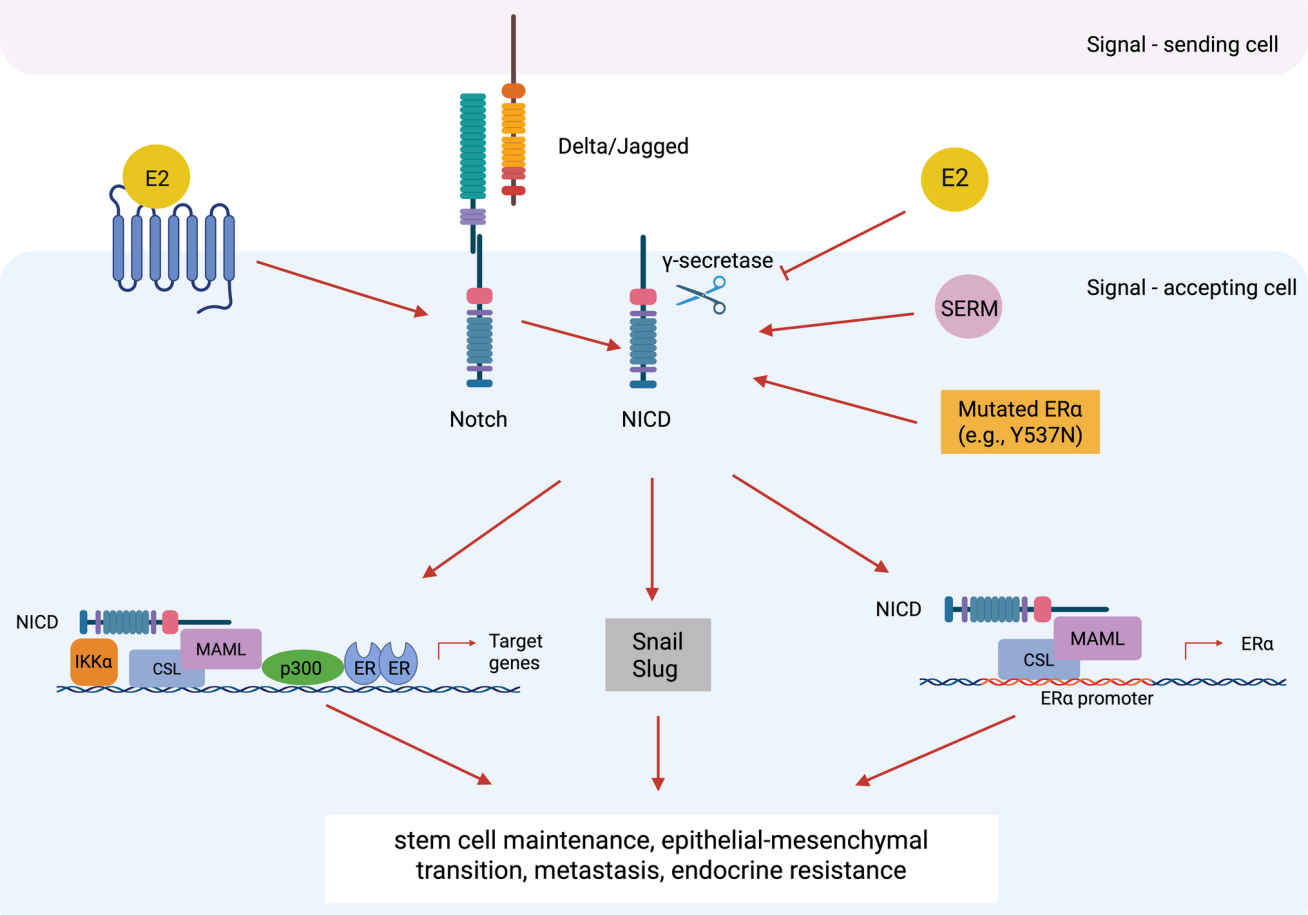
Schematic representation of ER-Notch signalling crosstalk in breast cancer. This figure highlights the molecular complexity of ER-Notch crosstalk in breast cancer. Crosstalk with ERα occurs through multiple mechanisms: (i) ERα promoter regulation by NICD-CSL-MAML; (ii) Notch-IKKα-CSL-MAML-driven recruitment of ERα to oestrogen-responsive element; (iii) oestradiol suppression of Notch by blocking γ-secretase-mediated cleavage; (iv) ERα mutations (e.g. Y537N, Y537S, D538G) detected in endocrine-resistant tumours activate Notch; (v) membrane ER crosstalk with Notch leads Snail/Slug driven epithelial-mesenchymal transition. ⊥, inhibition; ←, promotion; ↲, initiation.  Figure created using biorender.com

Notch signalling interacts closely with ER pathways in BCa. Oestrogen modulates multiple Notch pathway components in endothelial cells by activating Notch1/4, suppressing Notch2, and enhancing Dll4 under angiogenic conditions [67]. In ERα-positive BCa, oestradiol suppresses Notch1 by blocking γ-secretase-mediated cleavage, whereas ERα inhibition reactivates Notch1/4, supporting oestrogen-independent and tamoxifen-resistant growth (Fig. 5) [12]. Reporter assays revealed an inverse correlation between Notch1/4 activity and ERα, with higher signalling in MDA-MB-231 compared with MCF-7 and T47D cells [34]. In ERα-positive luminal BCas, Dll1 is highly expressed compared with normal and TNBC/basal subtypes, where ERα signalling stabilises Dll1 by preventing its ubiquitination and degradation. The elevation of Dll1 promotes proliferation, angiogenesis, BCSC maintenance, tumour growth, and metastasis and correlates with poorer survival outcomes, while its knockdown suppresses these processes [58]. Moreover, oestrogen activates a positive crosstalk between membrane ER (GPER) and Notch signalling, driving Notch/Snail-mediated EMT in both ERα-positive and ERα-negative BCa cells (Fig. 5) [68].

Different Notch receptors exert subtype-specific roles. Notch1 drives basal-like cell expansion in ERα-positive luminal cancers under endocrine therapy. Specifically, hormone treatment stimulates luminal tumour cells to give rise to ERα-negative basal-like cell progeny via Notch1 activation, whereas γ-secretase inhibition preserves hormone responsiveness [33]. In ERα-positive BCa models, Notch1 cooperates with ERα at the chromatin level via IKKα- and CSL-MAML-driven recruitment to oestrogen-responsive loci, promoting ligand-independent ERα target gene transcription. Importantly, dual inhibition of Notch and ERα signalling synergistically suppresses tumour growth (Fig. 5) [34].

Notch3 maintains the luminal phenotype in BCa by transcriptionally activating ERα, thereby suppressing EMT, tumour growth, and metastasis, while its loss promoted EMT and tumour progression [69]. Notch3 has been shown to enhance ERα expression by binding to the DNA-binding transcription factor CSL sites in ERα promoters, with Notch3 silencing leading to decreased ERα mRNA and protein levels in BCa cell lines (Fig. 5). Further, Notch3 correlates positively with ERα and GATA3, and suppresses EMT and metastasis via a Notch3–GATA3 axis [70]. However, in endocrine-resistant MCF-7 cells, Notch3 signalling is upregulated, inducing PBX1 and establishing transcriptional programmes that sustain resistance [7]. Chromatin reprogramming in resistant MCF-7 cells promoted enrichment and overexpression of Notch pathway and target genes, with Notch3 activation identified as a key driver of resistance [71].

Notch4 expression has been reported to be inversely correlated with hormone receptors in BCa patients and positively correlated with lymph node and tumour size [54]. Under classical ERα signalling, oestrogen induces canonical targets while repressing Notch pathway components (e.g. Notch4, HES1, and HEY1); however, endocrine therapies abrogate ERα activity, relieving this repression and leading to Notch activation, enrichment of Notch-high cancer stem cells, and therapy resistance [6]. Moreover, *ESR1* mutations (e.g. Y537N, Y537S, D538G), frequently detected in endocrine-resistant tumours, also appear to promote BCSC-like phenotypes through Notch4 activation, suggesting Notch blockade may mitigate endocrine resistance in *ESR1*-mutant tumours (Fig. 5) [6]. Anti-oestrogen treatments such as tamoxifen or fulvestrant suppress proliferation in ERα-positive BCa cells but simultaneously enrich the BCSC population via Notch activation (Jag1-Notch4), whereas co-inhibition of Notch prevents this stem cell increase and may forestall anti-oestrogen resistance [72]. In endocrine-resistant ERα-positive BCa, PKCα overexpression promotes tamoxifen-resistant oestrogen-independent growth by upregulating Notch4 via AP-1 [73]. In tamoxifen-resistant sub-lines of the MCF-7 BCa cell line, the Notch4-STAT3 signalling axis drives both EMT and BCSC maintenance, contributing to metastatic potential and therapeutic resistance [69]. Endocrine therapy-resistant MCF-7 cells have elevated Notch4 expression, accompanied by pronounced EMT characteristics. Interestingly, knockdown of Notch4 reverses the EMT phenotype and restores endocrine therapy sensitivity [34].

## Therapeutic implications of targeting Notch signalling in breast cancer

**Fig. 6:**
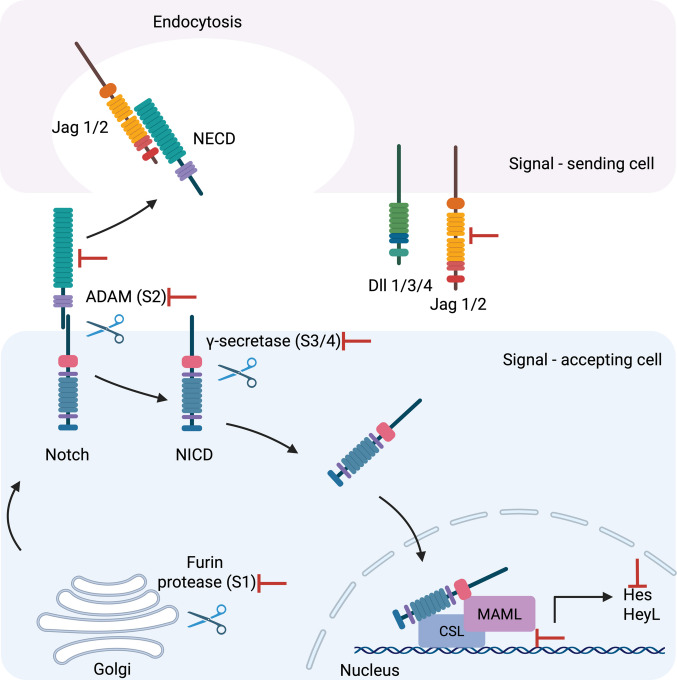
Targeted inhibition sites within the canonical Notch signalling pathways. This schematic highlights the multiple intervention points in the canonical Notch signalling cascade across both signal-sending and signal-accepting cells. Red bars indicate key inhibition points: Ligand or Notch receptor blockade (e.g. monoclonal antibodies); ADAM (S2) and γ-secretase (S3/4) inhibitors that prevent receptor activation; furin inhibitors (S1) that impair Notch maturation; nuclear complex disruption or transcriptional repression that prevents downstream gene activation. The figure was designed using biorender.com

Emerging evidence from preclinical and clinical studies supports the potential of targeting the Notch signalling pathway to overcome endocrine resistance in ERα-positive BCa, with inhibition of Notch signalling, particularly in combination with endocrine therapy, chemotherapy, or immunotherapy showing promise in reversing resistance and improving outcomes in advanced or treatment-refractory disease. The Notch signalling pathway can be targeted at different stages: cleavage by furin convertase (S1), cleavage by ADAM protease (S2), cleavage by gamma secretase (S3/4), ligand-receptor binding, NICD nuclear translocation, regulation of Notch target gene expression, downstream signalling and crosstalk, and targeting Notch receptor posttranslational modifications (Fig. 6, Table 1) [74].

**Table 1:** Therapeutic strategies and clinical trials focussing on Notch signalling in breast cancer.  Partial response (PR), Stable disease (SD), Complete response (CR), Progression-free survival (PFS), Overall survival (OS), Progressive disease (PD), Gastrointestinal (GI), Recommended phase II dose (RP2D), Maximal tolerated dose (MTD).

Trial ID	Agent	Node	Modality	Patient population	Evidence level	Salient efficacy	Key toxicity	Status
NCT01149356	RO4929097 and exemestane and goserelin acetate	Targeting S3/4 cleavage	γ-Secretase inhibitor and aromatase inhibitor and synthetic GnRH	ERα-positive BCa	Phase I	Among 14 patients, 1 achieved a PR and 7 had SD, no CR were observed. Median PFS was 3.2 months, and OS was 6 months	Nausea, anorexia, hyperglycaemia, hypophosphatemia, fatigue, cough	Terminated
NCT01158274	RO4929097 and capecitabine	Targeting S3/4 cleavage	γ-Secretase inhibitor and anti-metabolite (inhibitor of DNA synthesis)	HER2-negative metastatic BCa and other solid tumours	Phase I	3 patients achieved confirmed PR, and 9 had SD. 18 patients experienced PD	Hypophosphatemia, fatigue, diarrhoea, nausea, vomiting	Completed
NCT01217411	RO4929097 and radiotherapy	Targeting S3/4 cleavage	γ-Secretase inhibitor	Metastatic BCa and other cancers	Phase I/II	N/A	N/A	Terminated
NCT01238133	RO4929097 and carboplatin and paclitaxel	Targeting S3/4 cleavage	γ-Secretase inhibitor and DNA synthesis inhibitor and microtubule inhibitor	TNBC	Phase I	5 of 10 patients achieved a complete pathological response in the breast and axilla, and 3 had minimal residual disease in the breast	Sensory neuropathy, anaemia, fatigue, depression	Terminated
NCT01151449	RO4929097	Targeting S3/4 cleavage	γ-secretase inhibitor	Metastatic TNBC	Phase II	N/A	N/A	Terminated
NCT01071564	RO4929097 and vismodegib	Targeting S3/4 cleavage	γ-Secretase inhibitor and smoothened (Hedgehog) inhibitor	Metastatic BCa	Phase I	N/A	N/A	Terminated
NCT01208441	RO4929097 and letrozole	Targeting S3/4 cleavage	γ-Secretase inhibitor and aromatase inhibitor	Postmenopausal, stage II/III BCa	Phase I	N/A	N/A	Terminated
NCT01131234	RO4929097 and cediranib maleate	Targeting S3/4 cleavage	γ-Secretase inhibitor and VEGF receptor inhibitor	Advanced BCa	Phase I	N/A	N/A	Completed
NCT00645333	MK-0752 and docetaxel/pegfilgrastim	Targeting S3/4 cleavage	γ-Secretase inhibitor and microtubule inhibitor and colony stimulating factor	Metastatic BCa	Phase I/II	Among 24 participants evaluable for response. 11 achieved a PR, 9 had SD, and 3 experienced PD	Pneumonitis, hand-foot syndrome, liver function test elevation, diarrhoea	Completed
NCT00756717	MK-0752 and tamoxifen or letrozole	Targeting S3/4 cleavage	γ-secretase inhibitor and antioestrogen and aromatase inhibitor	Early-stage ERα-positive BCa	Phase IV	The combination of MK-0752 with endocrine therapy was feasible, safe, and well tolerated	Periorbital oedema/cough, nausea, and axillary paraesthesia, facial rash, fatigue	Completed
NCT00106145	MK-0752	Targeting S3/4 cleavage	γ-Secretase inhibitor	Metastatic/locally advanced BCa and other solid tumours	Phase I	Among patients with high-grade gliomas, 1 CR was observed, and 10 additional patients achieved SD	Fatigue, GI symptoms	Completed
NCT01295632	MK-0752 and MK-8669 (ridaforolimus)	Targeting S3/4 cleavage	γ-Secretase inhibitor and mTOR inhibitor	Advanced BCa and other tumours	Phase I	18 patients were evaluable for response, including 10 with HNSCC. 1 patient with HNSCC achieved a CR, another had a confirmed PR, and 3 SD	Stomatitis, diarrhoea, decreased appetite, hyperglycaemia, thrombocytopenia, asthenia, rash	Completed
NCT02299635	PF-03084014	Targeting S3/4 cleavage	γ-Secretase inhibitor	Metastatic TNBC	Phase II	N/A	N/A	Terminated
NCT02338531	PF-03084014	Targeting S3/4 cleavage	γ-Secretase inhibitor	Non-metastatic TNBC	Phase II	N/A	N/A	Withdrawn
NCT00878189	PF-03084014	Targeting S3/4 cleavage	γ-Secretase inhibitor	BCa and other advanced solid tumours	Phase I	150 mg twice daily was selected as RP2D. 1 patient with advanced thyroid cancer achieved a CR, and 5 patients with desmoid tumours achieved PR. SD was observed in 14 additional patients, including 2 with desmoid tumours	Diarrhoea, nausea, fatigue, hypophosphatemia, vomiting, rash, decreased appetite	Completed
NCT01876251	PF-03084014 and docetaxel	Targeting S3/4 cleavage	γ-Secretase inhibitor and microtubule inhibitor	Metastatic/locally advanced BCa	Phase I	1 patient had a confirmed PR for advanced TNBC. 6 patients had SD. The MTD was PF-03084014 100 mg BID/docetaxel 75 mg/m^2^	Neutropenia, fatigue, leukopenia, nausea, alopecia, diarrhoea, anaemia	Terminated
NCT01292655	AL101 (BMS-906024)	Targeting S3/4 cleavage	γ-Secretase inhibitor	BCa and other advanced/metastatic solid tumours	Phase I	SD was best response in 9 patients	Hypophosphatemia, diarrhoea, hypokalaemia, anaphylaxis, anaemia, AST increase, nausea, pruritus, vomiting	Completed
NCT01653470	AL101 (BMS-906024) and other drugs (paclitaxel, 5-fluorouracil, carboplatin, leucovorin, irinotecan)	Targeting S3/4 cleavage	γ-Secretase inhibitor and others	Advanced/metastatic BCa and other solid tumours	Phase I	N/A	N/A	Completed
NCT04461600	AL101	Targeting S3/4 cleavage	γ-Secretase inhibitor	Metastatic TNBC	Phase II	N/A	N/A	Terminated
NCT02784795	LY3039478 (crenigacestat) and other drugs (taladegib, abemaciclib, cisplatin, gemcitabine, carboplatin, LY3023414)	Targeting S3/4 cleavage	γ-Secretase inhibitor and others	Advanced/metastatic BCa (TNBC)	Phase I	The MTD was defined 25 mg in part B (with LY3023414) and 50 mg in part C (with abemaciclib) and was not established in part A (with taladegib). No CR or PR were observed, SD occurred in 6 patients in part B and 6 in part C, but none in part A	GI symptoms (diarrhoea, nausea, vomiting)	Completed
NCT01695005	Crenigacestat (LY3039478) and prednisone	Targeting S3/4 cleavage	γ-Secretase inhibitor (Notch 1) and corticosteroid	BCa and other advanced or metastatic cancers	Phase I	Part A (dose-escalation phase), part B served as the dose-confirmation phase. PR was observed in 1 patient with ERα-positive BCa in part A, not in part B, whereas SD was achieved in 17 patients in part A and 19 patients in part B	GI symptoms (diarrhoea vomiting)	Completed
NCT01778439	OMP-52M51 (brontictuzumab)	Inhibition of Notch receptors	Anti-Notch1	Refractory solid tumour	Phase I	Among 31 patients, 2 had PR, 10 SD, 24 PD. The MTD was 1.5 mg/kg every 3 weeks	Diarrhoea, fatigue, nausea, vomiting, AST increase	Completed
NCT02129205	PF-06650808	Inhibition of Notch receptors	Anti-Notch3	Advanced BCa	Phase I	5 patients achieved a PR, including 4 with ERα-positive BCa and 1 with TNBC. 16 patients achieved SD, including 8 of 14 with ERα-positive disease	Fatigue, decreased appetite, nausea, alopecia, abdominal pain, pruritus, vomiting	Terminated
NCT01277146	OMP-59R5	Inhibition of Notch receptors	Anti-Notch2/3	BCa and other solid tumours	Phase I	The MTD was 2.5 mg/kg weekly, and 7.5 mg/kg on every other and every 3-week schedules. No objective response. 9 patients had SD	GI toxicity (diarrhoea, fatigue, nausea, anorexia, and vomiting and abdominal pain, constipation)	Completed
NCT00744562	Demcizumab (OMP-21M18)	Inhibition of Notch ligands	Anti-Dll4	BCa and other advanced solid tumours	Phase I	Among 48 patients, SD was observed in 21, including tumour reductions across multiple tumour types. 16 patients at 10 mg/kg had evidence of stabilisation of disease or response	Hypertension, fatigue, anaemia, headache, nausea, hypalbuminaemia, dizziness, dyspnoea	Completed
NCT00871559	Enoticumab (REGN421)	Inhibition of Notch ligands	Anti-Dll4	BCa and other advanced solid malignancies	Phase I	Two PR (NSCLC bronchoalveolar subtype and ovarian cancer) and SD in 16 patients across multiple tumour types including 2 BCas. RP2D was 4 mg/kg every 3 weeks or 3 mg/kg every 2 weeks	Fatigue, nausea, vomiting, hypertension, headache, anorexia	Completed
NCT02298387	OMP-305B83	Inhibition of Notch ligands	Anti-Dll4/VEGF	BCa and other solid tumours	Phase I	Among 66 treated patients, 4 achieved a PR (notably in ovarian cancer) and 17 had SD	Hypertension, headache, fatigue, pulmonary hypertension	Completed
NCT03292783	ABL001	Inhibition of Notch ligands	Anti-Dll4/VEGF	Advanced solid tumour	Phase I	ABL001 was tolerated up to 7.5 mg/kg without significant treatment-related adverse events	Hypertension, anorexia, general weakness, headache, anaemia	Completed
NCT04492033	ABL001 and paclitaxel or irinotecan	Inhibition of Notch ligands	Anti-Dll4/VEGF and microtubule inhibitor/topoisomerase inhibitor	Advanced solid tumour	Phase I/II	9 patients achieved a PR. The median PFS were 9.4 months. 12-month OS rate was 53%	Neutropenia, hypertension, anaemia, thrombocytopenia	Terminated
NCT04714619	Limantrafin (CB-103) and anastrozole or letrozole	Transcription complex inhibitors	Inhibitor of Notch transcription activation complex (CSL-NICD) and AROMATASE inhibitors	Pre- and post-menopausal women with ERα-positive BCa	Phase II	N/A	N/A	Terminated
NCT03422679	Limantrafin (CB-103)	Transcription complex inhibitors	Inhibitor of Notch transcription activation complex (CSL-NICD)	Metastatic BCa and other tumours	Phase I/II	No objective responses were observed, although 37 patients achieved SD, including 23 with adenoid cystic carcinoma. Median OS for the solid tumour cohort was 9.2 months, and median PFS was 1.9 months	Laboratory abnormalities (elevated liver enzymes, amylase, or lipase), anaemia, visual changes	Terminated

### Targeting S1 cleavage

Inhibiting furin with its prodomain modulates calcium homeostasis in cancer cells via Orai and TRPC6 channels. This, in turn, suppresses malignant traits and sensitises cells to apoptotic stimuli. Loss of furin protease attenuated its downstream oncogenic pathways involving PI3K/AKT and MAPK/ERK1/2. Further, furin activity in T cells plays a critical role in modulating the tumour immune environment. Inhibition of furin was shown to reduce TNBC development and spread. PLAC1 acts as an oncogenic driver in BCa by engaging furin to activate NICD and suppress PTEN, thereby facilitating invasion and metastasis [12].

### Targeting S2 cleavage

The anti-cholesterol drug lomitapide has been shown to act as a dual inhibitor of TACE/ADAM17 and γ-secretase to suppress Notch signalling in TNBC. Its repurposing shows potential as a therapeutic strategy to impair tumour progression, reverse EMT, reduce stemness and metastatic features, and increase oxidative stress to promote cell death [75]. Targeting ADAM17 with the inhibitor TAPI-2 effectively suppresses the downstream oncogenic PI3K-AKT pathway in BCa [76]. *In vitro* results suggest that targeting ADAM–17 with D1(A12) antibody may offer promising anti-cancer activity against TNBC [77]. Another study highlights that targeting ADAM protease activity (GW280264X) may enhance immune surveillance by preventing the escape of BCa cells from immune detection [78].

### Targeting S3/4 cleavage

Targeting the S3/4 cleavage step of Notch activation with γ-secretase inhibitors (GSIs) has been extensively explored in BCa. RO4929097 (NCT01149356; NCT01158274; NCT01217411; NCT01238133; NCT01151449; NCT01071564; NCT01208441 and NCT01131234) has been evaluated in multiple phase I trials, alone or in combination with chemotherapy (exemestane, paclitaxel/carboplatin, gemcitabine, capecitabine) and targeted agents (cediranib) (Table 1). These studies demonstrated manageable toxicity, pharmacokinetic compatibility, and preliminary activity across solid tumours, including BCa [23, 79, 80].

Early phase I trials demonstrated tolerability of MK-0752 (NCT00106145), although common adverse effects such as diarrhoea, fatigue, and nausea were noted. In combination with docetaxel, MK-0752 (NCT00645333) showed clinical benefit and reduced BCSC markers in tumour biopsies (Table 1) [29]. Preclinical studies revealed that GSIs (MK-0752/RO4929097) inhibit tumour growth in Notch3⁺ BCa but may enrich BCSCs via IL-6 induction; this effect can be reversed by IL-6R blockade with tocilizumab [35].

PF-03084014 (nirogacestat; NCT01876251) has shown promising activity in advanced TNBC patients in phase I study, as both a monotherapy and in combination with docetaxel, reducing EMT, BCSC populations, and chemoresistance [29]. The study by Hossein et al. highlighted the combined inhibition of Notch (PF-03084014), AKT (MK-2206), or IKK/NF‑κB (Bay11-7082) as a potential strategy to disrupt stemness and therapeutic resistance [81]. In a phase I study of patients with advanced solid tumours, PF-03084014 (NCT00878189) demonstrated effective Notch pathway suppression and promising antitumour activity, supporting its further clinical development (Table 1) [82].

The phase I clinical trials evaluated crenigacestat (LY3039478; NCT02784795/NCT01695005) in combination with chemotherapeutic agents such as gemcitabine, cisplatin, and carboplatin. The combinations were poorly tolerated and demonstrated limited clinical efficacy in patients with advanced or metastatic solid tumours, including BCa [11]. Another clinical trial showed that patients receiving AL101 (NCT01292655) treatment had a sustained partial response with a maximal decrease in tumour size (Table 1) [83].

The GSI Begacestat (GSI-953) successfully inhibits BCa growth and increases programmed cell death. When combined with doxorubicin, these effects were substantially amplified, resulting in significantly higher rates of apoptosis in TNBC models [84]. Another GSI, MRK-003, significantly suppressed proliferation of MCF-7 and MDA-MB-231 BCa cells in a dose- and time-dependent manner [85]. Chemotherapy-induced senescence in BCa promotes EMT and metastasis by activating Notch1 signalling in neighbouring non-senescent cells, a non-autonomous effect that can be blocked by the GSI DAPT, highlighting the potential of combining Notch inhibition with chemotherapy to prevent relapse [86]. In TNBC, targeting γ-secretase with LY411575 has also been shown to improve anti-PD-1 efficacy by reducing tumour-associated macrophage infiltration and enhancing cytotoxic T-cell activity, while nanoparticle-based co-delivery of LY411575 and paclitaxel enabled spatially controlled Notch inhibition, reducing recurrence after surgery in murine models [87, 88].

Proteasome inhibitors having γ-secretase activity such as cbz-Leu-Leu-Nle-CHO, z-Leu-Leu-Nle-CHO, and Z-Ile-Leu-CHO have been shown to destabilise Notch and impair BCa progression [89]. Novel derivatives such as NMK-T-057 have also shown preclinical efficacy, inhibiting NICD1-HES1-Akt signalling, EMT, and BCSC traits with minimal toxicity [90].

Despite encouraging preclinical and early clinical findings, GSIs remain limited by non-specific blockade of all four Notch receptors, causing gastrointestinal and systemic toxicities (e.g. goblet cell metaplasia, impaired mucosal integrity), which constrain long-term use [35]. Consequently, more selective approaches, such as receptor- or ligand-specific antibodies, transcription complex inhibitors (e.g. CB-103), and novel delivery systems are being developed to enhance efficacy while mitigating toxicity.

### Inhibition of Notch receptors

Preclinical evidence supports antibody-based Notch inhibition. Monoclonal antibodies against Notch1’s negative regulatory region synergised with docetaxel to reduce mammosphere formation, BCSC frequency (CD44⁺/CD24⁻/low), and tumour recurrence in TNBC models [36]. In BCa models, anti-Notch1 antibody (23814) effectively inhibited ligand-mediated Notch1 signalling, suppressed angiogenesis, and reduced tumour growth without the gastrointestinal toxicity seen with broad Notch inhibition. When combined with VEGFR inhibition, it achieved synergistic antitumour activity [73].

Promising results have also been reported for brontictuzumab (OMP-52M51; NCT01778439), a monoclonal antibody selectively inhibiting Notch1. In a phase I trial of advanced solid tumours, including BCa, the antibody achieved a 17% objective response rate with a manageable safety profile (Table 1) [23].

Tarextumab (OMP-59R5; NCT01277146), targeting Notch2/3, was well tolerated in early-phase studies, and demonstrated disease stabilisation (Table 1) [29]. Combining EGFR (panitumumab) and Notch (tarextumab) inhibition using a bispecific antibody counters BCSC-mediated resistance in TNBC [24].

In patients with advanced BCa and other solid tumours, PF-06650808 (NCT02129205), an anti-Notch3 antibody–drug conjugate, was generally well tolerated with manageable adverse events and resulted in partial responses (particularly ERα-positive/HER2-negative BCa) and stable disease in over half of the treated cohort [91]. This underscores the therapeutic advantage of paralogue-specific antibodies over pan-Notch GSIs.

### Inhibition of Notch ligands

In a multicancer phase I trial, the anti-DLL4 antibody demcizumab (OMP-21M18; NCT00744562) was tolerated at doses less than 5 mg weekly, with hypertension, fatigue, anaemia, headache, nausea, hypoalbuminaemia, dizziness, and dyspnoea each occurring in more than 10% of patients. Among 48 evaluable patients, stable disease was achieved in 21, with tumour reductions observed across multiple tumour types (Table 1) [35]. Enoticumab (REGN421; NCT00871559), a fully human anti-Dll4 antibody, was similarly well tolerated in phase I testing with fatigue, nausea, vomiting, hypertension, headache, and anorexia most frequently reported, and infrequent cardiopulmonary events. Antitumour activity included two partial responses (NSCLC bronchoalveolar subtype and ovarian cancer) and stable disease in 16 patients across multiple tumour types including two BCas; the recommended phase II dose was 4 mg/kg every 3 weeks or 3 mg/kg every 2 weeks [74, 92]. Navicixizumab (OMP-305B83; NCT02298387), a bispecific Dll4/VEGF antibody, showed manageable toxicity in phase I evaluations. Among 66 treated patients, four achieved a partial response (notably in ovarian cancer) and 17 had stable disease, with hypertension, headache, fatigue, and pulmonary hypertension representing the most frequent treatment-related adverse events, supporting further combination studies [93]. Another bispecific Dll4/VEGF antibody, ABL001 (NCT03292783/NCT04492033) in phase I/II clinical trials has been implemented in combination with chemotherapy agents like paclitaxel on advanced solid tumours (human gastric or colon cancer), inhibited tumour progression compared to each monotherapy (Table 1) [24, 73]. In addition, DL1.72 and IgG-69, anti-Dll1 antibodies, inhibited proliferation, migration, mammosphere formation, and stemness features in ERα-positive BCa models [56, 94]. Jag blockade has also shown promise therapeutically. Selective Jag1 inhibition reduced BCSC populations and impaired TNBC growth, with dual targeting of tumour- and stroma-derived Jag1 suppressing brain metastases and restoring blood–brain barrier integrity [95]. The humanised antibody 15D11, targeting Jag1, markedly reduced bone metastatic burden and osteolysis in preclinical models of BCa and was found to synergise with chemotherapy [10].

### Transcription complex inhibitors

CB-103, an orally active small-molecule inhibitor that disrupts the NICD-CSL-MAML transcriptional complex, offers a downstream alternative to γ-secretase inhibition with reduced intestinal toxicity. In the first-in-human phase I/II study, CB-103 (NCT03422679) demonstrated a manageable safety profile. No objective responses were observed, although 37 of 76 patients achieved stable disease, including 23 of 40 with adenoid cystic carcinoma. Median overall survival for the solid tumour cohort was 9.2 months, and median progression-free survival was 1.9 months at a median follow-up of 5.4 months. The recommended phase II dose was 500 mg twice daily on a 5-days-on/2-days-off schedule and treatment-related adverse events included laboratory abnormalities (elevated liver enzymes, amylase, or lipase), anaemia, and visual changes. In this trial, CB-103 demonstrated limited antitumour activity as monotherapy. In the phase II trial (NCT04714619), CB-103 was evaluated with aromatase inhibitors in luminal BCa, particularly ESR1-mutant endocrine-resistant cases (Table 1). Preclinical studies also demonstrated synergy between CB-103 and fulvestrant or paclitaxel, reducing mammosphere formation and suppressing tumour growth in ESR1-mutant xenografts [96]. Other transcription complex inhibitors include IMR-1, which blocks MAML1 recruitment to the NICD-CSL complex, showing preclinical efficacy and synergy with the Wnt inhibitor PRI-724 that disrupts NICD-CSL-MAML1 assembly, thereby directly repressing Notch target gene transcription [97].

### Downstream signalling modulators and multi-targeted natural and small molecular compounds

A diverse range of natural and synthetic compounds has been reported to suppress BCa progression through modulation of Notch signalling, often converging on proliferation, survival, EMT, angiogenesis, and BCSC maintenance. The Withaferin-A analogue ASR490 binds the Notch1 NRR, suppressing tumour growth, EMT, and BCSC [98]. Similarly, 6-Shogaol (a derivative from ginger) downregulates Notch target genes *HES1* and *CCND1* (cyclin D1), reducing cell proliferation in MCF-7 BCa cells. In MDA-MB-231 cells, resveratrol reduced the expression of *Notch1*, *Dll4*, *Jag1*, and *HES5* at the transcript level and significantly suppressed Notch1 and Dll4 protein levels. Notch1-Dll4 signalling is a driver of proliferation through regulation of genes such as *CCND1* and *MYC*; its downregulation by resveratrol correlated with decreased cell viability.

Oridonin, naturally derived from *Isodon rubescens*, inhibits proliferation, angiogenesis, EMT, and survival pathways by targeting Notch1-4 signalling. Another natural compound, cimigenoside, blocks γ-secretase activity by targeting the catalytic subunit PSEN-1, which prevents NICD from binding to CSL and thereby disrupts Notch signalling; in parallel, it promotes apoptosis by downregulating Bcl-2. Similarly, ZQL-4c suppresses Notch-AKT signalling, induces ROS, and triggers G₂/M arrest [90]. In line with these effects, natural products such as 3-O-(E)-p-coumaroylbetulinic acid inhibit γ-secretase and Notch1 activation, inducing E-cadherin expression, whilst downregulating BCL2, survivin, and cyclin D1 levels, and suppressing mammosphere formation in TNBC cells. Compounds including moricin, celastrol, triptolide, psoralidin, genistein, selenite, and others have all been reported to attenuate Notch signalling and exhibit significant anticancer activity. Notably, many of these natural compounds are pleiotropic and modulate multiple signalling pathways, with Notch representing one of several affected nodes; moreover, the majority of supporting data remain preclinical, necessitating careful pharmacological characterisation and translational validation prior to clinical application [99–101].

## Conclusion

The Notch signalling pathway is essential for normal mammary development and tissue homeostasis and, when dysregulated in specific oncogenic contexts, contributes to BCa initiation, progression, and therapy resistance. Its crosstalk with ERα is particularly significant in hormone receptor-positive disease, where endocrine therapy or oestrogen deprivation can paradoxically activate Notch and foster resistance. In ERα-negative disease, Notch signalling is closely associated with cancer stemness, epithelial mesenchymal transition, and angiogenesis, with increasing evidence for roles in tumour-immune interactions. Consequently, rational combinations targeting Notch alongside endocrine or chemotherapeutic agents may enhance efficacy and delay resistance. Although numerous Notch-directed agents have entered clinical testing, none has yet achieved regulatory approval due to toxicity and specificity challenges. Emerging approaches such as receptor-/ligand-specific antibodies, transcription-complex inhibitors (e.g. CB-103) and rational combination therapies aim to improve selectivity and clinical benefit.

## Data Availability

No datasets were generated or analysed during the current study.
